# The Use of Cyclodextrin Inclusion Complexes to Increase the Solubility and Pharmacokinetic Profile of Albendazole

**DOI:** 10.3390/molecules28217295

**Published:** 2023-10-27

**Authors:** Yili Ding, Zhiyuan Zhang, Charles Ding, Shufeng Xu, Zhe Xu

**Affiliations:** 1 College of Science and Technology, Wenzhou-Kean University, Wenzhou 325000, China; 2 Wenzhou Municipal Key Laboratory for Applied Biomedical and Biopharmaceutical Informatics, Wenzhou-Kean University, Wenzhou 325060, China; 3 Zhejiang Bioinformatics International Science and Technology Cooperation Center, Wenzhou-Kean University, Wenzhou 325060, China; 4 Dorothy and George Hennings College of Science, Mathematics and Technology, Kean University, 1000 Morris Ave, Union, NJ 07083, USA; 5 Life Science Department, Foshan University, Foshan 528000, China; 6 Keck School of Medicine of USC, Los Angeles, CA 90089, USA

**Keywords:** albendazole, methyl-*β*-cyclodextrin, inclusion complex, water solubility, in vitro and in vivo PK study

## Abstract

Albendazole is the preferred deworming drug and has strong insecticidal effects on human and animal helminth parasites, showing remarkable activity against hepatocellular carcinoma and colorectal cancer cells. However, it is classified as being in class II in the Biopharmaceutics Classification System due to its poor water solubility (0.2 mg/L) and high permeability, which make the clinical application of albendazole impractical. Through complexation with methyl-*β*-cyclodextrin, as the best result so far, albendazole’s water solubility was increased by 150,000 times, and albendazole could be 90% released during the first 10 min. In an in vivo pharmacokinetic study, the *C*_max_ and *T*_max_ of the active metabolized sulfoxide were changed from 2.81 µg/mL at 3 h to 10.2 µg/mL at 6 h and the AUC_0–48_ was increased from 50.72 h⁎μg/mL to 119.95 h⁎μg/mL, indicating that the inclusion complex obtained can be used as a new oral therapeutic anti-anthelmintic and anti-tumor agent formulation.

## 1. Introduction

Albendazole has a broad anthelmintic spectrum, strong insecticidal effect on human and animal helminth parasites, low toxicity, and good safety, and was recommended by the World Health Organization as the preferred deworming drug [1]. Furthermore, albendazole is a microtubule depolymerizing agent with remarkable activity against hepatocellular carcinoma and colorectal cancer cells in vitro and in vivo [2].

Albendazole belongs to class II in the Biopharmaceutics Classification System due to its poor water solubility (0.2 mg/L) and high permeability [3]. Albendazole’s low intestinal absorption rate, low blood concentration in the body, and low bioavailability required a high dosage for treating systemic helminth infections; the current clinical cure rate for non-intestinal parasitic diseases is only about 30% [4]. The clinical application of the drug in cancer using IP or IV routes may require a much higher aqueous solubility, usually 5–10 mg/mL. This is because low concentrations of drugs in solution would require very large volumes to reach the required therapeutic dose, which made the application of albendazole impractical, and its clinical usefulness as an anti-cancer agent is severely hampered [5].

**Figure 1:**
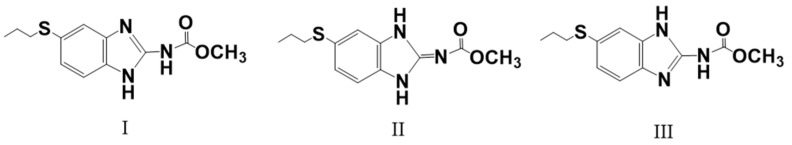
Three tautomeric forms of albendazole.

Since 1991, numerous attempts have been made to improve albendazole’s solubility and thereby enhance its efficacy, such as the use of soybean oil emulsion [6], surfactants [7], liposomes [8], polyvinylpyrrolidone [9], ionization in acids [10], and complexations with cyclodextrins. Among these methods, complexation with cyclodextrins is better than the others, since albendazole presents three tautomeric forms, I, II, and III, as shown in Figure 1 [11]. Cyclodextrins can form inclusion complexes with them to prevent their interconversion. Forms I and II are enantiomerically related forms [12], and the commercially available form I is metastable at room temperature while form II is stable. Therefore, complexation with cyclodextrins can not only increase solubility, but also stabilize the compounds [13].

A *β*-Cyclodextrin inclusion complex has been reported to stabilize albendazole and, consequently, improve its water solubility and dissolution rate. However, this complexation only increased the solubility 53.4 times due to *β*-cyclodextrin’s low aqueous solubility (18 mg/mL). The solid product of the complexation was analyzed using solid NMR spectroscopy, however, signals from albendazole in the complex were not observed in the spectrum [14,15].

The solubility of albendazole in a complex with HP-*β*-cyclodextrin was increased from 2 to 10,000 fold to reach a solubility as high as 1.9 mg/mL [16,17,18,19,20,21]. Adding citric acid, ascorbic acid, hydrochloric acid, or acetic acid to the aqueous solution of HP-*β*-cyclodextrin could increase the solubility of albendazole from 0.2 µm/mL to 1.5 mg/mL [22,23,24]. When albendazole was stirred with 40% of sulfobutylether-*β*-cyclodextrin in water at 25 °C with a pH of 2.3 for 3 days, its water solubility reached 8 mg/mL. Under the same conditions, HP-*β*-cyclodextrin could make its water solubility reach 6.4 mg/mL [25]. However, as albendazole is soluble in a 0.1 HCl solution (0.4 mg/mL), it was hard to say whether the 8 mg/mL solubility was due to the acidic condition or due to cyclodextrin’s solubilization capability [26].

A variety of techniques such as FTIR, DSC, TAG, X-ray, and SEM have been used to confirm the formation of complexes in the solid state [27]. However, an increased water solubility can only be confirmed by its proton NMR spectrum in D_2_O.

When albendazole was treated with 40% of HP-*β*-cyclodextrin, sulfobutylether-*β*-cyclodextrin, or methyl-*β*-cyclodextrin in water, its aqueous solubility was remarkably increased from 0.2 μg/mL to 0.79 mg/mL, 1.17 mg/mL, and 1.52 mg/mL, respectively, and these increased water solubilities were confirmed by proton NMR spectra in D_2_O [28,29,30].

Citrate-*β*-cyclodextrin, itaconyl-*β*-cyclodextrin, and succinyl-*β*-cyclodextrin were synthesized and complexed with albendazole via spray drying. The drug dissolution rate and water solubility were improved slightly, and their proton NMR spectra in 0.1N DCl in D_2_O were recorded. Since albendazole is soluble in a 0.1 HCl solution (0.4 mg/mL), the chemical shifts from albendazole in the complex in the NMR spectrum could not fully support the drug’s water solubility [31,32].

A trace amount of water-soluble polymer such as polyvinylpyrrolidone K30 was added to the cyclodextrin complex to increase the drug’s solubility [33,34,35].

Recently, the co-precipitation of *β*-cyclodextrin and albendazole using a supercritical antisolvent technique in a miscible solution of DMSO, acetone, and CO_2_ produced the precipitate as microparticles. After characterization using spectroscopic, thermal, and crystallographic analyses, this new solid form was found to contain tautomer II of albendazole. The dissolution rate was increased about four times in comparison to that of albendazole alone due to the formation of the inclusion complex and a synergetic effect between both components. However, the water solubility increase was not measured and confirmed [36]. It was reported that the cyclodextrin’s solubilization capability in a complexation with albendazole can be enhanced by adding bile salt [37].

Overall, even with great efforts towards preparing albendazole/cyclodextrin complexes, the water solubility has still not been significantly improved and is far away from the 5–10 mg/mL that is required for a cancer therapeutic dose. In the continuation of an interest in drug inclusion complexes [38,39,40,41,42,43,44,45,46,47], there was a further study on improving the water solubility of albendazole through the formation of inclusion complexes with cyclodextrins. In this paper, new results are reported on albendazole’s water solubility through complexation with methyl *β*-cyclodextrin, where the water solubility of albendazole was increased from 0.2 μg/mL to 30 mg/mL.

## 2. Results and Discussion

UV spectra of the solutions of albendazole in the mixture of acetic acid and ethanol (1:19, 0.1 mg/mL) and cyclodextrins in water (1 mg/mL) were recorded from 200 nm to 400 nm, and the maximum absorption of albendazole at 295 nm was observed. Cyclodextrins have no UV absorption around this wavelength, and 295 nm was selected for a HPLC analysis of albendazole. A series of albendazole solutions in methanol with concentrations of 0.01, 0.02, 0.04, 0.06, 0.08, and 0.1 mg/mL were analyzed using HPLC, and, based on the concentrations and peak areas, the regression equation was obtained as Y = 15598X + 10.71 (R^2^ = 0.9991, Y = concentration, and X = peak area).

The HPLC standard curves and a sample analysis including plasma samples were performed in the same HPLC instrument with the same C-18 column over a short period of time to avoid systematic errors, before and after the HPLC analysis, and the standard solution of ketoconazole was checked using HPLC to confirm the stability of the HPLC analysis conditions.

**Figure 2:**
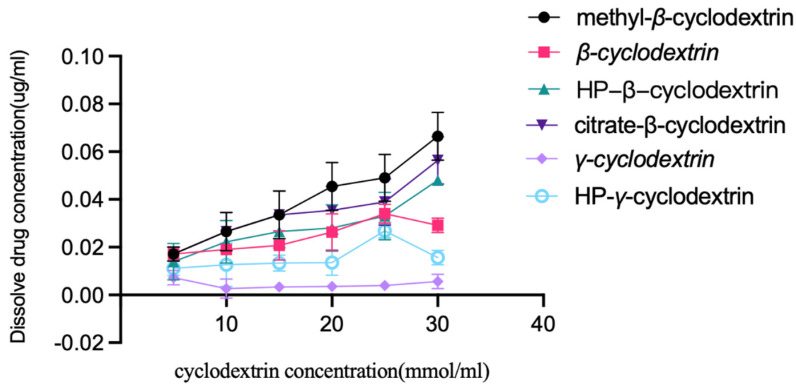
Solubility of albendazole in water solutions of *β*-cyclodextrin, HP-*β*-cyclodextrin, methyl-*β*-cyclodextrin, citrate-*β*-cyclodextrin, *γ*-cyclodextrin and HP-*γ*-cyclodextrin with different concentrations.

Extra albendazole was added to the solutions of *β*-cyclodextrin, HP-*β*-cyclodextrin, methyl *β*-cyclodextrin, HP-*γ*-cyclodextrin *γ*-cyclodextrin, and citrate-*β*-cyclodextrin in water with different concentrations (5, 10, 15, 20, 25, and 30 mmol/mL). After stirring at room temperature for 48 h, each mixture was filtered, diluted with water, and checked using HPLC; the results are summarized in Figure 2. It was found that methyl-*β*-cyclodextrin had the best solubilization effect on albendazole, and the linear relationship between albendazole and methyl-*β*-cyclodextrin was classified as A_L_ type according to Higuchi and Connors’s description [48].

By using the ultrasonic method, aqueous solution stirring method, hydrothermal reactor, microwave method, and CO_2_ supercritical method [49], methyl-*β*-cyclodextrin was complexed with albendazole. The resulting solutions were analyzed using HPLC, and it was found that the ultrasonic method could provide the complex with the largest increase in water solubility.

The reaction temperature, reaction time, ratio of albendazole and methyl-*β*-cyclodextrin, and ultrasonic power affected the complexation. Through single-factor and orthogonal strategies based on the Design of Experiments approach [50], many complexation conditions were designed to form the complexes with methyl *β*-cyclodextrin based on changing the ratio, ultrasonic power, reaction temperature, and reaction time. After the HPLC analysis, the best condition for increased solubility was found as follows: the ratio should be 1:3, the reaction time should be 45 min (every 5 s reaction with 10 s intervals), the ultrasonic power should be 80%, and the reaction temperature should be 25 °C with occasional ice cooling. The best solubility was found as high as 27 mg/mL.

Through self-association, cyclodextrins and their complexes can form aggregates or micelle-like structures to solubilize water-insoluble drugs by non-inclusion complexation [51], and water-soluble polymers can enhance the solubilizing effect of cyclodextrins [52]. Small amounts (0.25%) of ten water-soluble polymers or organic salts were added to albendazole’s complexation reaction, respectively. After workup and HPLC analysis, it was found that complexation with the addition of sodium dodecyl sulfate could increase albendazole’s water solubility to as high as 30 mg/mL, which is 150,000 times better than that of the drug alone and the best result so far. This complex has a 16% inclusion rate and 86% inclusion yield, and was used for complexation confirmation in in vitro and in vivo pharmacokinetic studies.

Only a trace amount of sodium dodecyl sulfate (0.25% based on the amount of albendazole) existed in the complex; the FTIR, DSC, and NMR spectra of the complex could not exhibit its characteristic signals.

**Figure 3:**
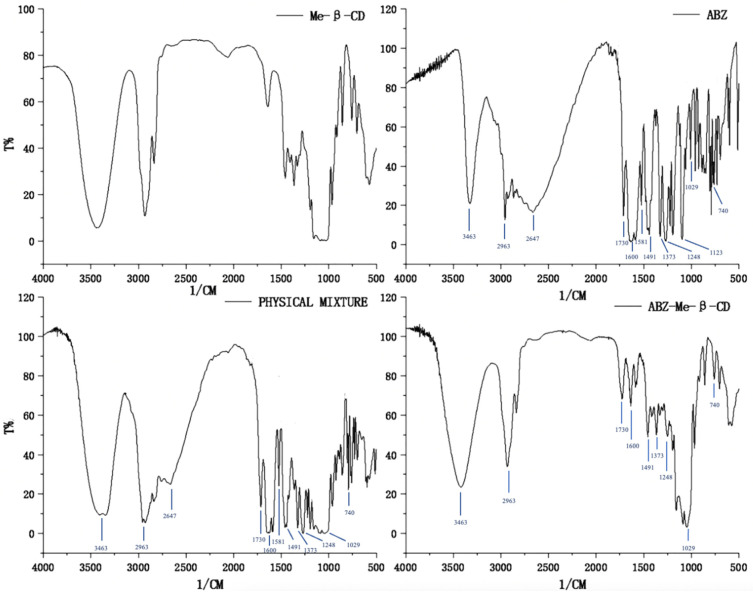
Fourier-transform infrared spectra of methyl-*β*-cyclodextrin, albendazole, the physical mixture of methyl-*β*-cyclodextrin and albendazole, and their inclusion complex.

FTIR spectra were used to confirm the complex of albendazole with random methyl-*β*-cyclodextrin. The FTIR spectra of methyl-*β*-cyclodextrin, albendazole, the physical mixture of albendazole and methyl-*β*-cyclodextrin, and their inclusion complex are shown in Figure 3. Compared to the spectrum of methyl-*β*-cyclodextrin, the characteristic peaks of albendazole appeared at 3463 cm^−1^ (N-H stretching in carbamate), 2963 cm^−1^ (C-H aliphatic stretching vibration), 2647 cm^−1^ (-NH intramolecular in imidazole), 1730 cm^−1^ (bending vibration of C=O bond in carbamate), 1600 cm^−1^ (C=C aromatic and N-H out of the plane bending in benzimidazole), 1581 cm^−1^ (stretching vibration of C=N group), 1491 cm^−1^ and 1373 cm^−1^ (C-N and C-O stretching vibrations), 1248 cm^−1^, 1123 cm^−1^, and 1029 cm^−1^ (CH and NH in plane bending vibrations and CH deformation), and 1000–600 cm^−1^ (skeletal vibrations, CH out of plane bending, NH_2_ rocking, and C-S stretching vibrations), and they are obviously seen in the physical mixture of albendazole and methyl-*β*-cyclodextrin, but are slightly reduced in the inclusion complex. These changes in the characteristic peaks of albendazole confirmed that the albendazole was complexed with cyclodextrin.

**Figure 4:**
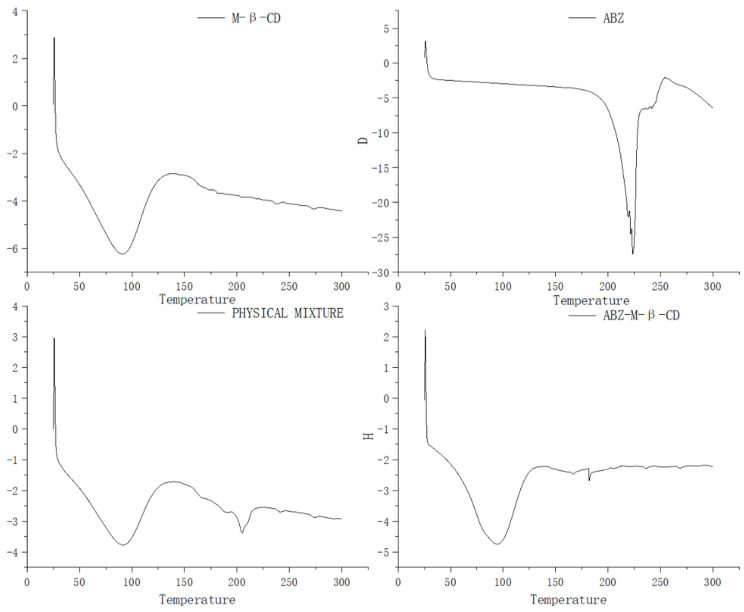
Differential scanning calorimetry curves of methyl-*β*-cyclodextrin, albendazole, the physical mixture of methyl-*β*-cyclodextrin and albendazole, and the inclusion complex.

The differential scanning calorimetry curves of albendazole, methyl-*β*-cyclodextrin, their physical mixture, and inclusion complex were recorded and are shown in Figure 4. The endothermic event in curve for methyl-*β*-cyclodextrin corresponding to dehydration from the cavity was seen in the range of 50–125 °C, in curve for albendazole, the endothermic event at 225 °C was seen due to its melting effect, in curve for the physical mixture of methyl-*β*-cyclodextrin and albendazole, the endothermic event in the range of 50–125 °C was seen for the dehydration of methyl-*β*-cyclodextrin, and the absence of the endothermic event at 225 °C corresponding to albendazole’s melting and the presence of an endothermic event at 205 °C suggested an interaction between albendazole and methyl-*β*-cyclodextrin in the physical mixture. In the curve of the inclusion complex, the same thermal profile as that of methyl-*β*-cyclodextrin was presented, probably due to the extra methyl-*β*-cyclodextrin in the complex. The characteristic endothermic peak of albendazole at 225 °C disappeared due to the loss of the crystalline structure caused by encapsulation, which confirmed the interaction between albendazole and methyl-*β*-cyclodextrin and the formation of a new phase.

**Figure 5:**
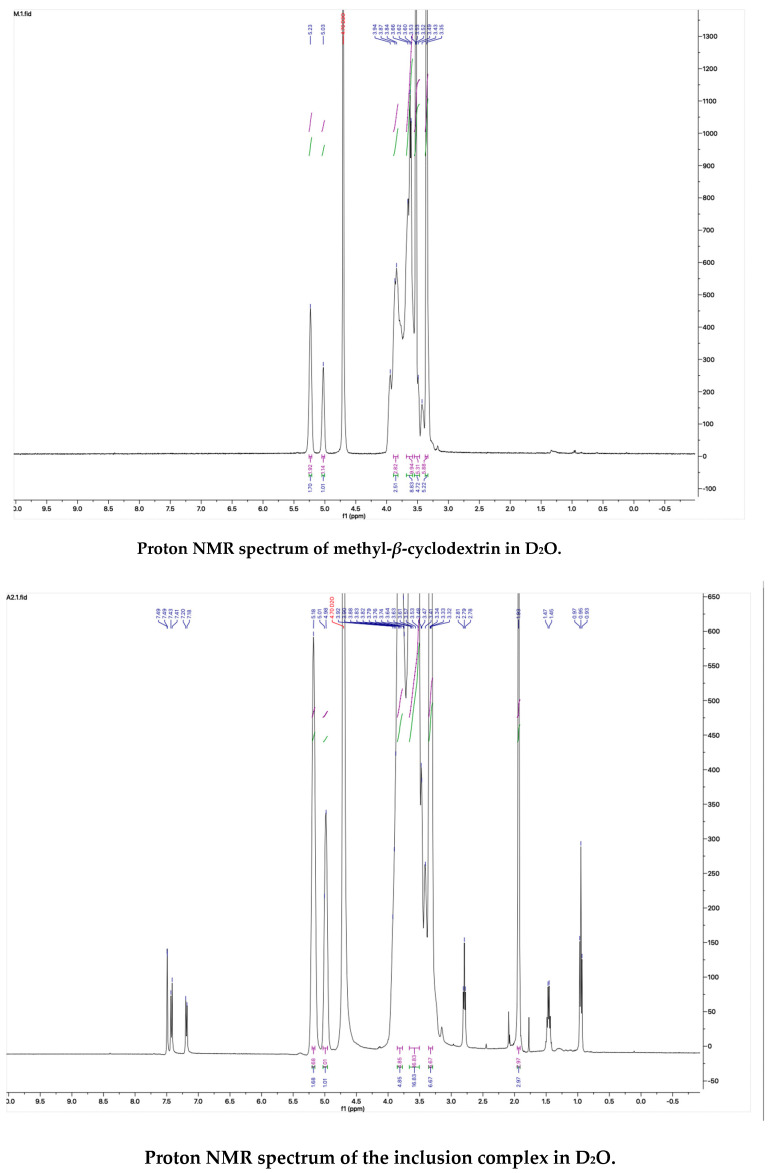

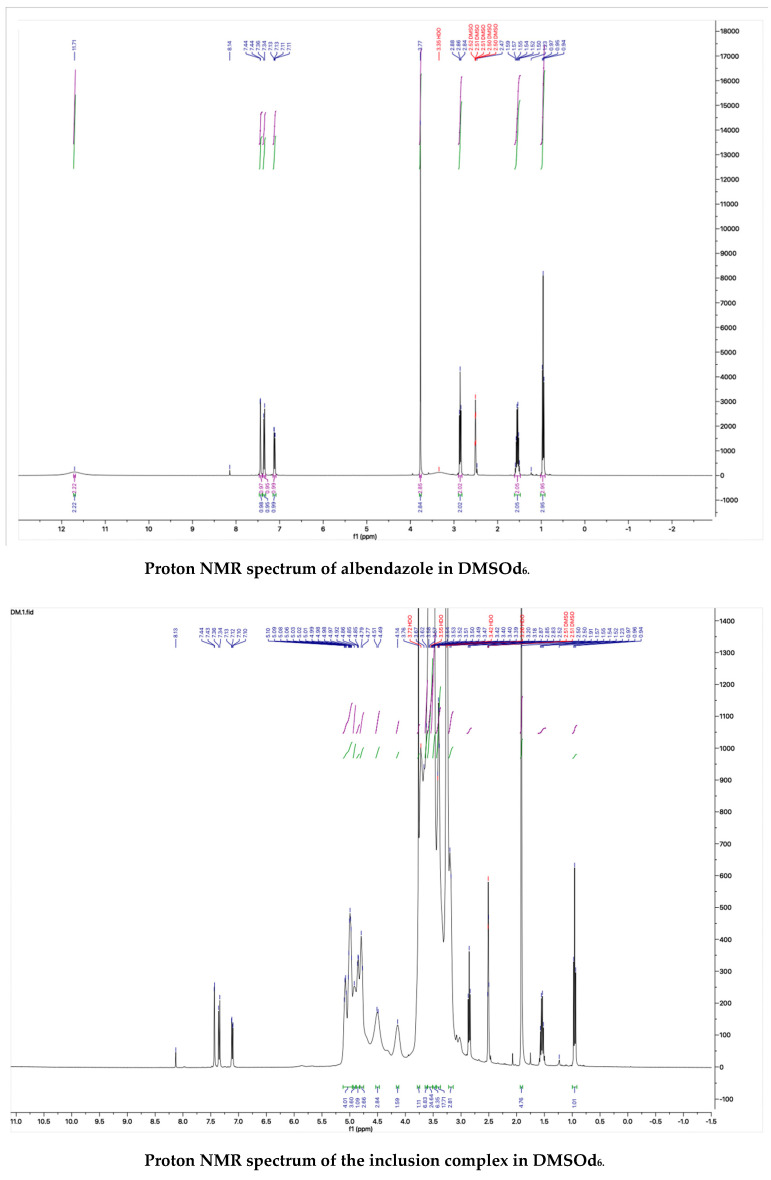
The proton NMR spectra of methyl-*β*-cyclodextrin and the inclusion complex in D_2_O, and the proton NMR spectra of albendazole and the inclusion complex in DMSOd_6_.

**Figure 6:**
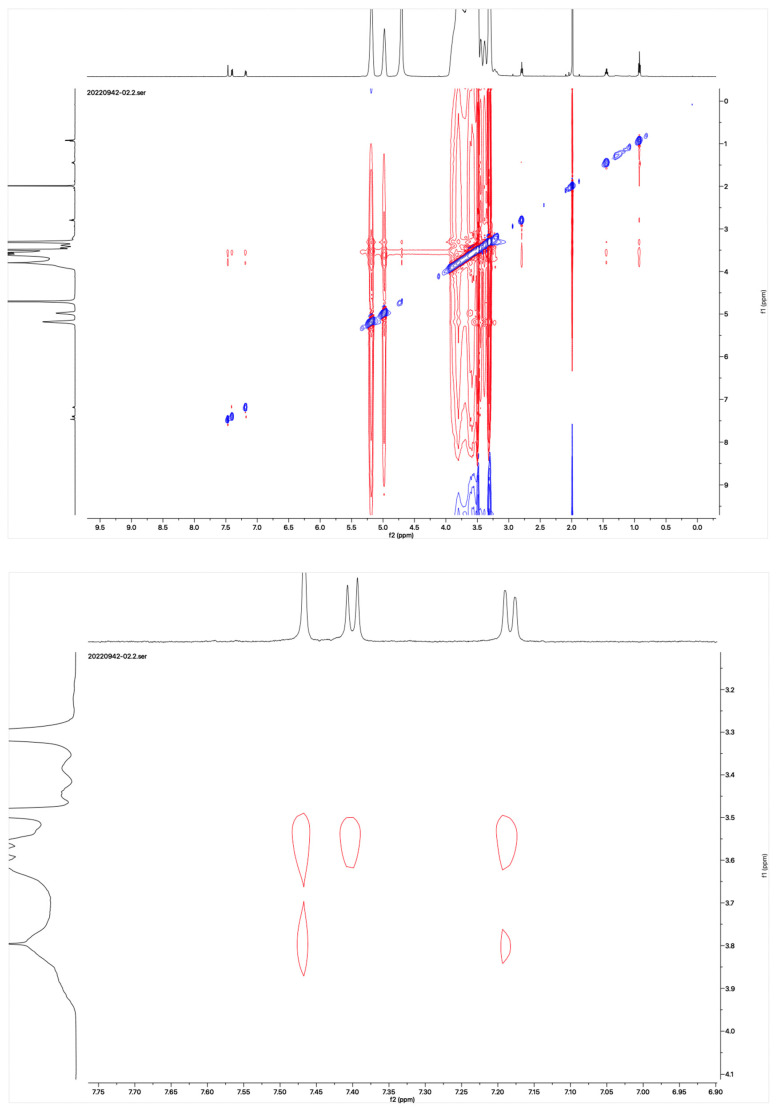

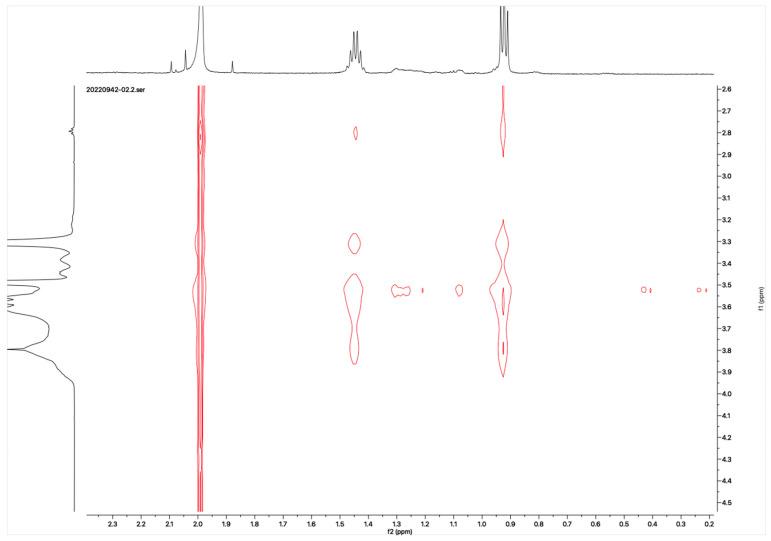
Roesy spectrum of the albendazole/methyl-*β*-cyclodextrin inclusion complex in D_2_O.

The proton NMR spectra of albendazole in DMSOd_6_, methyl-*β*-cyclodextrin in D_2_O, and the inclusion complex in D_2_O and DMSOd_6_ are shown in Figure 5. The proton NMR spectrum of the complex in D_2_O exhibited the signals from albendazole at 7.61 (1H, s), 7.56 (1H, d, *J*_1_ = 8.0 Hz), 7.33 (1H, d, *J*_1_ = 8.0 Hz), 2.91 (2H, t, *J*_1_ = 8.0 Hz), 1.54 (2H, m), and 1.06 (3H, t, *J*_1_ = 8.0). The signals of OCH_3_ overlapped with the signals from methyl-*β*-cyclodextrin in the range from 4.00 ppm to 3.50 ppm. The proton NMR spectrum of albendazole in DMSOd_6_ exhibited signals at 7.46 (1H, s), 7.34 (1H, d, *J*_1_ = 8.0 Hz), 7.11 (1H, d, *J*_1_ = 8.0 Hz), 3.76 (3H, s, OCH_3_), 2.85 (2H, d, *J*_1_ = 8.0 Hz), 1.53 (2H, d, *J*_1_ = 8.0 Hz), and 0.95 (3H, d, *J*_1_ = 8.0 Hz); the proton NMR data of albendazole in the complex in DMSOd_6_ exhibited signals at 7.42 (1H, s), 7.34 (2H, d, *J*_1_ = 8.0 Hz), 7.10 (2H, d, *J*_1_= 8.0 Hz), 2.65 (2H, d, *J*_1_ = 8.0 Hz), 1.54 (2H, d, *J*_1_ = 8.0 Hz), and 0.95 (3H, d, *J*_1_ = 8.0 Hz). The chemical shift difference in the signals of albendazole in DMSOd_6_ between the complex and the drug alone could not support the formation of the inclusion complex. The Roesy spectrum of the inclusion complex in D_2_O was recorded (Figure 6). The signals from the three aromatic protons, two methylene, and one methyl protons showed the interactions with methyl-*β*-cyclodextrin, which confirmed the formation of the inclusion complex.

**Figure 7:**
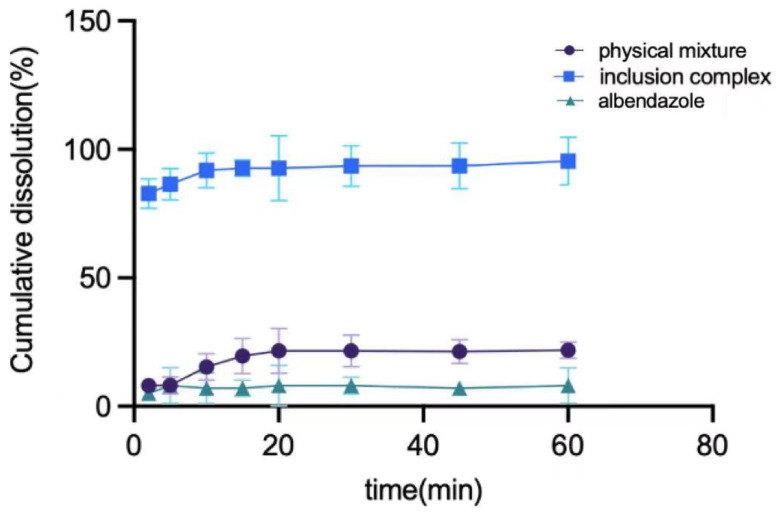
The dissolution curves of albendazole, the physical mixture of albendazole and methyl-*β*-cyclodextrin, and their inclusion complex.

The in vitro PK study of albendazole, the physical mixture of albendazole and methyl-*β*-cyclodextrin, and the complex are summarized in Figure 7. The albendazole in the complex can be 90% released in 10 min, which is 14 times better than that of albendazole alone and 5 times better than that of the physical mixture.

**Figure 8:**
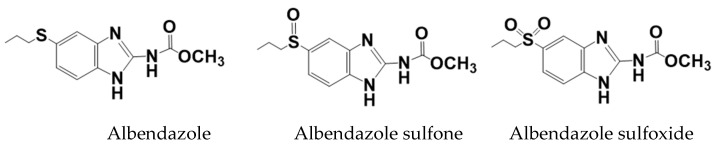
Molecular structures of albendazole, albendazole sulfone, and albendazole sulfoxide.

Albendazole is rapidly metabolized in the liver to albendazole sulfoxide and albendazole sulfone [53], and their structures are shown in Figure 8. Albendazole sulfoxide has excellent anthelmintic activity [54]; therefore, the sulfoxide is mostly found in the blood, and hence has been primarily characterized in the PK studies conducted to date [55].

**Figure 9:**
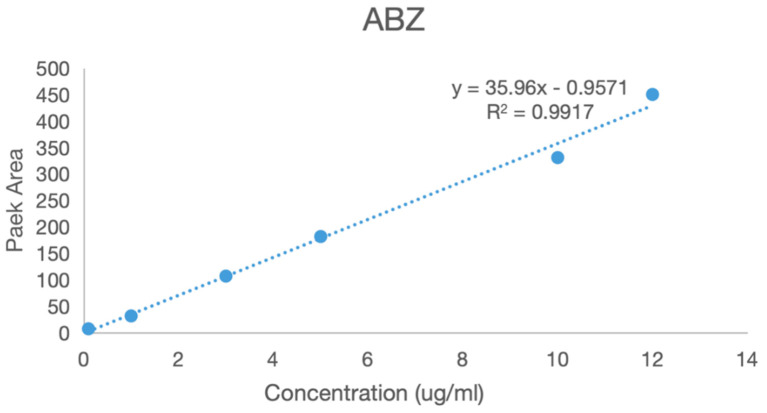

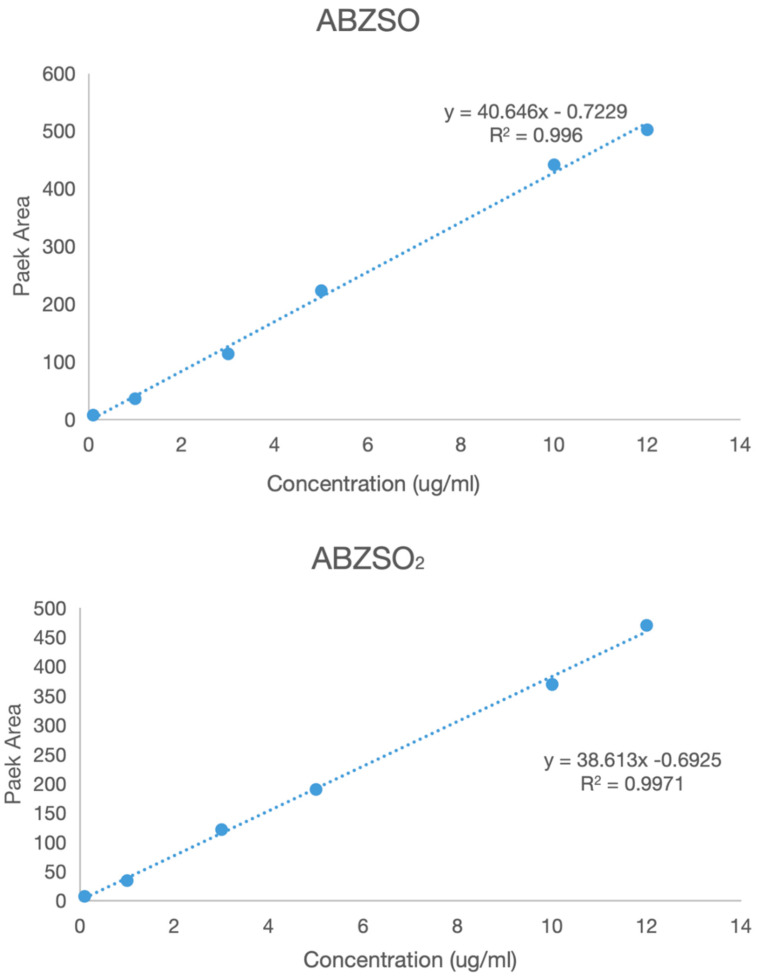
The standard curves of albendazole, albendazole sulfone, and albendazole sulfoxide in blank dog plasma.

A series of solutions of albendazole, albendazole sulfone, and albendazole sulfoxide in blank plasma with different concentrations were analyzed using HPLC in the range from 0.1 μg/mL to 5 μg/mL. The peak areas in HPLC and the concentrations of albendazole in the plasma solutions had a linear relationship, and the standard curve equations were obtained for albendazole as: Y = 35.96X − 0.9571 (coefficient of determination R^2^ = 0.9917, Y = peak area, X = concentration, S/N ≥ 10, LLOD = 0.054 μg/mL, LLOQ = 0.165 μg/mL), for albendazole sulfoxide as: Y = 40.464X − 0.7299 (coefficient of determination R^2^ = 0.996, Y = peak area, X = concentration, S/N ≥ 10: LLOD = 0.043 μg/mL, LLOQ = 0.141 μg/mL), and for albendazole sulfone as: Y = 38.613X − 0.6925 (coefficient of determination R^2^ = 0.9971, Y = peak area, X = concentration, S/N ≥ 10: LLOD = 0.044 μg/mL, LLOQ = 0.147 μg/mL). Their standard curves are shown in Figure 9.

**Figure 10:**
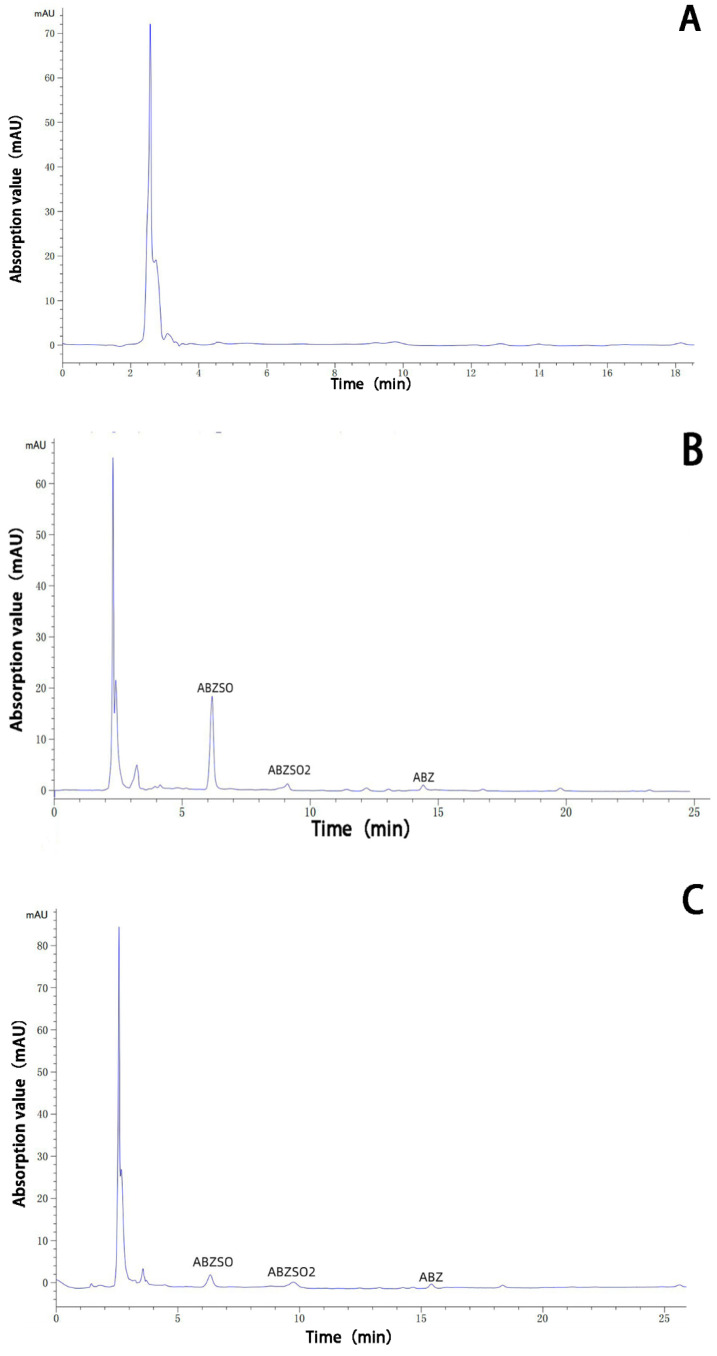

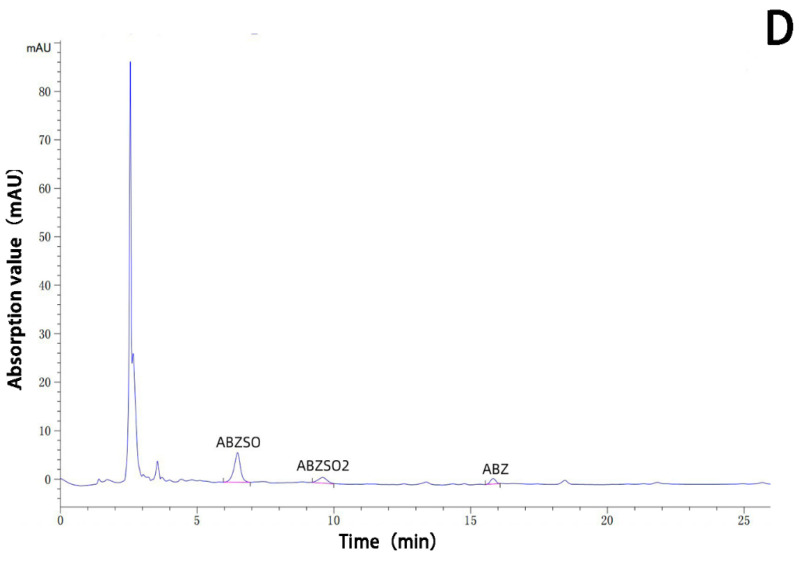
HPLC analysis of the sample of the blank dog plasma (**A**), the dog plasma containing albendazole, albendazole sulfoxide, and albendazole sulfone (**B**), the plasma from dogs dosed with albendazole (**C**), and the plasma from dogs dosed with the complex (**D**).

The albendazole, albendazole sulfoxide, and albendazole sulfone in blank dog plasma and the blank dog plasma were analyzed using HPLC and are shown in Figure 10. Under the HPLC conditions we used, the blank dog plasma did not interfere with the detection of albendazole, albendazole sulfoxide, and albendazole sulfone in the dog’s plasma.

The recovery rate and intra-assay coefficient of variation of the variations in albendazole, albendazole sulfoxide, and albendazole sulfone in the blank plasma with concentrations of 2 μg/mL, 5 μg/mL, and 10 μg/mL were determined and summarized as follows:

recovery rateintra-assay coefficient of variationAlbendazole90.2 ± 6.1% to 109.4 ± 4.8% 2.2% to 3.62%Albendazole sulfoxide89.8 ± 3.6% to 117.2 ± 2.2% 1.81% to 2.24%Albendazole sulfone93.9 ± 4.1% to 103.2 ± 3.1% 2.78% to 5.49%

**Figure 11:**
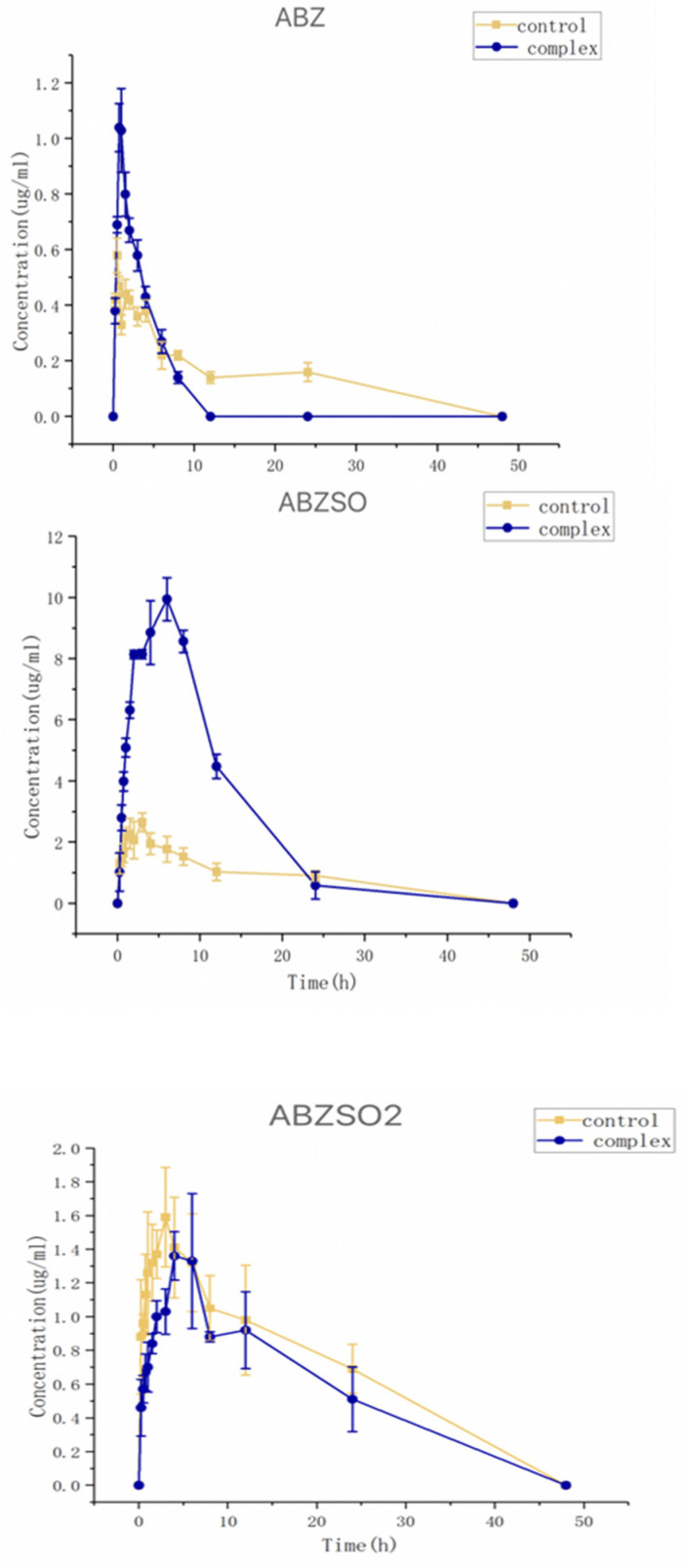
Curves of concentrations of albendazole, albendazole sulfone, and albendazole sulfoxide in plasma from dogs administered with albendazole or its methyl-*β*-cyclodextrin complex.

The collected plasma samples from dogs dosed with albendazole or its complex were analyzed using HPLC. The relationships between the concentrations of albendazole, albendazole sulfoxide, or albendazole sulfone in the dog plasma and time are shown in Figure 11. The pharmacokinetic parameters (mean ± SD) were calculated by using a noncompartmental analysis by Phoenix WinNonlin software (version 8.3) and are summarized in Table 1.

After oral administration, the albendazole was partially metabolized to sulfoxide and albendazole sulfone, and the albendazole sulfoxide was the anthelmintic active substance. The *C*_max_ and *T*_max_ of the albendazole sulfoxide in the dog plasma were found to be 10.18 μg/mL at 6.0 h for the complex group and 2.81 μg/mL at 3.0 h for the albendazole group. Through complexation, the *C*_max_ of albendazole sulfoxide was increased by 3.62 times, and the delayed *T*_max_ could require less frequent dosing within the clinically effective therapeutic range. The AUC_0–48_ of albendazole sulfoxide changed from 0.72 h⁎μg/mL in the albendazole group to 119.95 h⁎μg/mL in the complex group to make the relative bioavailability as high as 236%; the *C*_max_ of albendazole was increased by 2.11 times (from 0.51 μg/mL at 0.5 h to 1.08 μg/mL at 0.75 h) and the AUC_0–48_ of albendazole was decreased from 9.92 h⁎μg/mL to 3.92 h⁎μg/mL; and the *C*_max_ of albendazole sulfone was decreased from 1.63 μg/mL at 4 h to 1.16 μg/mL at 4 h and the AUC_0–48_ of albendazole sulfone was decreased from 34.66 h⁎μg/mL to 22.50 h⁎μg/mL.

Through complexation with methyl-*β*-cyclodextrin, the AUC_0–48_ of the active substance albendazole sulfoxide in vivo was doubled, the *C*_max_ was more than tripled, and the *T*_max_ was doubled, indicating that the inclusion complex of albendazole with methyl-*β*-cyclodextrin is the best formulation so far of albendazole as a potential anti-anthelmintic and anti-tumor agent.

## 3. Methods and Materials

Methyl-*β*-cyclodextrin (average molecular weight: 1303), hydroxypropyl-*γ*-cyclodextrin, albendazole, albendazole sulfoxide standard (98%), and albendazole sulfone (98%) were obtained from Shanghai Macklin, Shanghai, P. R. China; citric acid, HP-*β*-cyclodextrin, and *β*-cyclodextrin were obtained from Sa’en Chemistry Technology, Guangzhou, Guangdong, P. R. China; anhydrous sodium acetate was purchased from Xilong Science Co., Ltd., Shantou, Guangdong, P. R. China; dogs were obtained from Guangdong Medical Laboratory Animal Center, Guangzhou, Guangdong, P. R. China; blank dog plasma was obtained from Guangzhou Rui-Te Co., Ltd., Guangzhou, Guangdong, P. R. China; low-fat dog foods were obtained from Shanghai Jibai Chong Industrial Co., Ltd., Shanghai, P. R. China.

### 3.1. Albendazole’s UV Maximum Absorbance Wavelength Determination

The solution of albendazole (10 mg) in acetic acid (5 mL) was diluted with ethanol (95%, 95 mL), 5 mL of the solution was diluted with ethanol (95%, 45 mL), and the resulting solution was used for UV (ultraviolet visible spectrophotometer, J51903001 from Shanghai Jinghua Technology Instrument Co., Ltd., Shanghai, P. R. China) scanning in the range of 200–400 nm.

### 3.2. Establishment of Albendazole HPLC Standard Curve

A series of methanolic solutions of albendazole at concentrations of 0.01 mg/mL, 0.02 mg/mL, 0.04 mg/mL, 0.06 mg/mL, 0.08 mg/mL, and 0.1 mg/mL were analyzed using HPLC (LC-15C high-performance liquid chromatograph from Shimadzu Enterprise Management Co., Ltd., Kyoto, Japan) with a UV detector (295 nm) on a Shim-pack VP-ODS C18 column (250 mm × 4.6 mm) using acetonitrile/water (30:70, *v*/*v*) as a mobile phase with a 1.0 mL/min flow rate. Based on the peak areas in the HPLC peaks and the concentrations, the linear regression equation and HPLC standard curve were obtained.

### 3.3. Preparation of Citrate-β-cyclodextrin

Citrate-*β*-cyclodextrin was prepared by using the procedure reported in the literature [56].

### 3.4. Solubility of Albendazole in Different Cyclodextrin Solutions

Excess albendazole was added to the solutions of *β*-cyclodextrin, HP-*β*-cyclodextrin, methyl-*β*-cyclodextrin, citrate-*β*-cyclodextrin, *γ*-cyclodextrin, or HP-*γ*-cyclodextrin in water at 5, 10, 15, 20, 25, and 30 mmol/mL concentrations. Each solution was stirred at room temperature for 48 h, filtered, diluted with water, and analyzed using HPLC.

### 3.5. Preparation of the Inclusion Complexes of Albendazole with Methyl-β-cyclodextrin

*Method using sonication*: the solution of albendazole (150 mg) in acetic acid (5 mL) was added to the solution of methyl-*β*-cyclodextrin (750 mg) in water (10 mL) with stirring uing a magnetic stirrer (RCT digital was obtained from IKA). The resulting solution was ultrasonicated in an ultrasonic reactor (JY92-IIN from Ningbo Xinzhi Biotechnology Co., Ltd., Ningbo, Zhejiang, China) at 25 °C and 520 W for 1 h with occasional iced water cooling, refrigerated at 4 °C for a few hours, evaporated, dissolved in water (15 mL), filtered through a 0.22 µm filter membrane. and freeze-dried at around −45 °C under a pressure of 32 pa for 48–72 h to give a clathrate solid powder.

*Solution method*: the solution of albendazole (150 mg) in acetic acid (5 mL) was added to the solution of methyl-*β*-cyclodextrin (750 mg) in water (10 mL). The mixture was stirred at 50 °C and 800 rpm for 6 h, refrigerated at 4 °C for several hours, evaporated, dissolved in water (15 mL), filtered through a membrane (0.22 µm), and freeze-dried at around −45 °C under a pressure of 32 pa for 48–72 h to give the clathrate solid powder.

*Using an autoclave*: the mixture of albendazole (150 mg) and methyl-*β*-cyclodextrin (750 mg) in water and acetic acid (30 mL, 67:33) was stirred at 120 °C and 800 rpm for 8 h in a high-temperature and pressure reaction kettle (BZ-100ML/SC-L from Shanghai Baikal Technology Group Co., Ltd., Shanghai, China), evaporated, added to water, filtered, and freeze-dried at around −45 °C under a pressure of 32 pa for 48–72 h to give the product.

*Using a microwave reactor*: the solution of albendazole (150 mg) in acetic acid (5 mL) was mixed with the solution of methyl-*β*-cyclodextrin (750 mg) in water (10 mL), and the resulting mixture was stirred at 300 rpm in a microcomputer microwave chemical reactor (WBFY-201 from Gongyi Yuhua Instrument Co., Ltd., Xinxiang, Henan, China) for 1 h. During the reaction, every 10 min of heating was followed by 5 min. of no heating. After refrigeration at 4 °C for several hours and evaporation, the residue was dissolved in water (15 mL), filtered (0.22 µm), and freeze-dried at around −45 °C under a pressure of 32 pa for 48–72 h to provide the solid powder.

*Using supercritical CO_2_*: the solution of albendazole (150 mg) in glacial acetic acid (5 mL) was mixed with the solution of methyl-*β*-cyclodextrin (750 mg) in water (10 mL), pumped with liquid CO_2_ in the reactor (BZ-100ML/S0-L from Baikal Shanghai Intelligent Technology Co., Ltd., Shanghai, China), and stirred at 25 °C with a pressure of 6.5 Mpa for 12 h. After the pressure was slowly released, the solution was kept in a refrigerator at 4 °C for a few hours, the solvent was removed via rotary evaporation, the residue was dissolved in water (15 mL), and, after filtration through a 0.22 μm filter membrane and freeze-drying at around −45 °C under a pressure of 32 pa for 48–72 h, the product was obtained as a solid powder.

### 3.6. The Preparation of the Physical Mixture of Albendazole and Methyl-β-cyclodextrin

The grounded albendazole and methyl-*β*-cyclodextrin were mixed (1:3 molar ratio) and passed through the 80-mesh sieve to provide the physical mixture.

### 3.7. Preparation of the Inclusion Complexes with Addition of Trace Amount of Water-Soluble Polymer or Organic Salt

The solution of albendazole (150 mg) in acetic acid (5 mL) was added to the solution of methyl-*β*-cyclodextrin (750 mg) in water (10 mL), 5.6 mg of water-soluble polymer or organic salts (0.25% of albendazole) was added, and after stirring for 30 min, the resulting solution was ultrasonicated at 25 °C and 520 W for 40 min with occasional ice cooling, refrigerated at 4 °C for a few hours, evaporated, dissolved in water (15 mL), filtered through a 0.22 µm filter membrane, and freeze-dried at around −45 °C under a pressure of 32 pa for 48–72 h to give a clathrate solid powder. The inclusion complex from the ultrasonic method with the addition of a trace amount of sodium dodecyl sulfate was used for the FTIR, DSC, NMR, and in vitro and in vivo PK study.

### 3.8. Fourier-Transform Infrared Spectroscopy Study

The dried samples of albendazole, methyl-*β*-cyclodextrin, their physical mixture, and inclusion complex were ground in an agate mortar, respectively, on a VERTEX 70 Fourier transform infrared spectrometer (From Bruker company, Leipzig, Germany). Each of them was mixed with spectrally pure potassium bromide under an infrared lamp, poured into a compression mold, and pressed to provide the KBr pellets for the Fourier-transform infrared spectroscopy analysis in the range of 400–4000 cm^−1^.

### 3.9. Differential Scanning Calorimetry Study

The differential scanning calorimetry curves of albendazole, methyl-*β*-cyclodextrin, their mixture, and inclusion compound were recorded on a DSC 214 differential scanning calorimeter (from Netzsch, Bobingen, Germany) by placing them in an aluminum crucible, heating at a rate of 10 °C/min in the scan range from 25 to 300 °C under a nitrogen atmosphere (150 mL/min). Al_2_O_3_ was used as a reference.

### 3.10. Proton NMR Study

The proton NMR spectra of the solutions of methyl-*β*-cyclodextrin, its inclusion complex with albendazole in D_2_O, the solutions of albendazole, and its inclusion complex in DMSOd_6_ were recorded on a Bruker spectrometer (400 MHz). The Roesy spectrum of the complex recorded in D_2_O was recorded on a Bruker spectrometer (600 MHz).

### 3.11. Determination of the Albendazole Content in the Complex

The inclusion complex (100 mg) was dissolved in water (1 mL) with stirring for 2 h, and the solution was diluted with water to the range of 10 μg/mL–100 μg/mL (the albendazole HPLC standard curve was established in this range). Based on the HPLC analysis, the albendazole content in the complex was calculated using the regression equation.

### 3.12. Determination of Water Solubility of Albendazole in the Complex

An excess amount of the inclusion complex was dissolved in water (1 mL) for 2 h to obtain a saturated aqueous solution; after filtration, the sample was ready for HPLC analysis.

### 3.13. Inclusion Rate and Inclusion Yield Determination

The inclusion rate and yield of the inclusion compound were calculated by using the methods shown below:

Inclusion yield (%) = [albendazole inclusion complex (mg)/added albendazole (mg) + methyl-*β*-cyclodextrin (mg)] × 100%

Inclusion ratio (%) = [albendazole in inclusion complex (mg)/added albendazole (mg)] × 100%.

### 3.14. Dissolution Rate Determination

The paddle method in Chinese Veterinary Pharmacopoeia 2010 Edition I was adopted, albendazole (50 mg), the albendazole inclusion compound (containing 50 mg of albendazole), and the physical mixture of albendazole and methyl-*β*-cyclodextrin (containing 50 mg of albendazole) in degassed 0.1 mol/L hydrochloric acid (900 mL) were stirred at 50 r/min at 37 ± 0.5 °C, respectively, on an RC-3 dissolution meter (basket USP Apparatus 1 from Tianjin Xintianguang Analytical Instrument Technology Co., Ltd., Tianjin, China). In total, 5 mL was taken from each of the mixtures at 1, 3, 5, 10, 15, 30, 45, 60, and 75 min, followed by the addition of 5 mL of the same dissolution medium at the same times. After filtration through a 0.22 μm microporous membrane for 30 s, the sample was analyzed using HPLC at a wavelength of 295 nm, and, based on the HPLC data, the dissolution rate curves were obtained.

### 3.15. In Vivo Pharmacokinetic Studies

Wenzhou-kean university approved the study and the ID of the ethics approval is issued as WKULAEC2023-00.

Twelve healthy adult dogs (half males and half females, 5 ± 0.1 kg) were grouped as the albendazole group and the complex group, weighed, numbered, fed with low-fat food and normal drinking water in a warm and ventilated environment for 1 week, fasted for one day, and orally dosed with albendazole or albendazole complex in water (25 mg/kg for albendazole), respectively.

Blood samples (2 mL) were collected through the forearm vein after the administration of the drugs at 0, 0.25, 0.5, 0.75, 1, 1.5, 2, 3, 4, 6, 8, 12, 24, and 48 h, transferred immediately to a heparinized test tube, centrifuged at 2000 rpm for 10 min, and stored at −20 °C in a freezer.

The thawed plasma sample (0.5 mL) was pipetted into a centrifuge tube (2 mL), added with sodium thiosulfate (200 μg) and ethyl acetate (1 mL), vortexed for 5 min, centrifuged (13,000 rpm) for 10 min, and transferred to a test tube. After repeated vortexing and centrifugation, the supernatant in the test tube was blown at 40 °C in a nitrogen blower to dryness, added with methanol (0.5 mL), vortexed for 5 min, centrifuged (13,000 r/min) for 10 min, and filtered with a 0.22 μm microporous membrane for HPLC analysis.

### 3.16. Standard Curves for Albendazole, Albendazole Sulfoxide, and Albendazole Sulfone in Plasma

The blank plasma (0.5 mL) in a plastic centrifuge tube was added to the solutions of albendazole, albendazole sulfoxide, or albendazole sulfone at concentrations of 5 μg/mL, 1 μg/mL, 0.5 μg/mL, 0.1 μg/mL, 0.05 μg/mL, and 0.025 μg/mL, vortexed and vibrated, extracted with ethyl acetate or dichloromethane, blown with nitrogen, dissolved in methanol (1 mL), and analyzed using high-performance liquid chromatography at 295 nm using a Shim-packVP-ODS C18 chromatographic column (250 L × 4.6 mm) to provide the standard curves.

### 3.17. Statistical Analysis

Each experiment was performed in triplicate, and the results were expressed as the mean ± standard deviation. The statistical analysis was performed through a one-way analysis of variance followed by Tukey’s honestly significant difference test using DPS software. Statistical significance was defined as *p* < 0.05.

## 4. Conclusions

The inclusion complex of albendazole with methyl-*β*-cyclodextrin prepared by using the ultrasonic method with the addition of a trace amount of sodium dodecyl sulfate could increase the water solubility of albendazole by around 150,000 times compared to albendazole alone. In its in vivo pk study, the *C*_max_ and *T*_max_ of the active metabolized substance sulfoxide was changed from 2.81 μg/mL at 3 h to 10.2 μg/mL at 6 h, and the AUC_0–48_ was increased from 50.72 h⁎μg/mL to 119.95 h⁎μg/mL, indicating that this inclusion complex can be used as a new potent oral therapeutic anti-anthelmintic and anti-tumor agent.

## Figures and Tables

**Table 1:** Pharmacokinetic parameters of albendazole, albendazole sulfoxide, and albendazole sulfone (mean ± SD) in albendazole group and complex group.

		Albendazole		
Parameter	Unit	ABZSO	ABZSO_2_	ABZ
*T*_max_	h	3.00 ± 1.15	4.00 ± 1.06	0.50 ± 0.21
*C*_max_	μg/mL	2.81 ± 0.67	1.63 ± 0.42	0.51 ± 0.19
*T*_1/2_	h	18.48 ± 1.13	14.66 ± 1.54	14.43 ± 2.56
AUC_0–48_	h⁎μg/mL	50.72 ± 6.28	34.66 ± 4.91	9.29 ± 2.53
MRT_0–48_	h	24.35 ± 1.42	21.55 ± 1.52	24.75 ± 4.13
		Complex		
Parameter	Unit	ABZSO	ABZSO_2_	ABZ
*T*_max_	h	6.00 ± 1.29	4.00 ± 0.44	0.75 ± 0.63
*C*_max_	μg/mL	10.20 ± 1.91	1.16 ± 0.61	1.08 ± 0.28
*T*_1/2_	h	3.04 ± 1.07	10.18 ± 1.09	1.88 ± 0.53
AUC_0–48_	h⁎μg/mL	119.95 ± 11.7	22.50 ± 3.61	3.92 ± 2.06
MRT_0–48_	h	7.70 ± 1.16	15.53 ± 1.42	3.43 ± 1.32

## Data Availability

Not applicable.

## Sample Availability

Samples of the compounds are available from the authors.
